# Impact of Covid-19 pandemic on trajectories of patients with severe alcohol use disorder treated with disulfiram

**DOI:** 10.1038/s41598-025-90081-5

**Published:** 2025-02-17

**Authors:** Maximilian Pilhatsch, Max Schallenberg, Diana Vogel-Blaschka, Ariana-Berenike Treu, Johannes Petzold, Lena Zander, Maik Spreer

**Affiliations:** 1 Department of Psychiatry and Psychotherapy, University Hospital Dresden, TUD Dresden University of Technology, Dresden, Germany; 2 Department of Psychiatry and Psychotherapy, Elblandklinikum Radebeul, Radebeul, Germany

**Keywords:** Pandemic, Environmental risk factor, Disulfiram, Antabuse, Linear trend analysis, Alcohol use disorder, Addiction, Risk factors

## Abstract

The manifestations and progression of alcohol use disorder (AUD) are influenced by a number of contextual factors, with the current coronavirus pandemic being a significant example. This pandemic has profoundly impacted nearly all aspects of human life and has, therefore, strongly influenced patients suffering from AUD. In some cases, the pandemic has led to a reduction in severity, while in others, it has had the opposite effect. In our own work we have been investigating the negative impact of the pandemic on 45 patients with AUD who were undergoing outpatient treatment, including supervised use of disulfiram (Antabuse), in a close-knit program. A linear trend analysis demonstrated significant alterations in the retention rate over a 3-year period, encompassing the pre-pandemic, pandemic, and post-pandemic periods. During the pandemic the number of treatment cancellations virtually increased. Following the pandemic, a tendency towards the normalization of patient numbers was observed. Our data indicate a high level of vulnerability among patients with severe AUD and highlight a need for the development of alternative, possibly telemedical, treatment methods.

## Introduction

The global pandemic of the novel coronavirus (COVID-19) has had a profound impact on various aspects of human life, affecting not only the general population but also previously marginalized or at-risk groups in particular. One of the areas severely impacted is alcohol consumption and associated diseases.

A higher mortality rate for COVID-19 infection has been observed in people with alcohol use disorder (AUD) compared to the general population^1^. It is important to distinguish between the direct associations of coronavirus infections and alcohol consumption, on the one hand, and indirect associations (e.g. due to environmental effects of lockdowns on alcohol consumption), on the other. Among various mechanisms discussed, chronic alcohol consumption increases the expression of genes such as Ace2, which is responsible for the production of the ACE2 protein. ACE2 is known to act as a gateway for coronaviruses, including the SARS-CoV-2 virus that causes COVID-19^2^. The elevated expression of ACE2 in alcohol abusers is likely to increase their susceptibility to coronavirus infection and mortality^2,3^. However, the indirect effects of pandemic restrictions on the trajectories of AUD have proven to be more complex and seem somewhat contradictory^2,4^. Some studies have reported regional differences in alcohol consumption during the pandemic, which may be influenced by the consideration of alcohol as an “essential” (e.g., the United States and the United Kingdom) or “banned” (e.g., India and South Africa) item with constraints leading to a decrease in overall consumption. In some regions, alcohol consumption increased by more than fourfold, while in other regions it remained unchanged^4^. Upon closer examination, it appears that the alcohol consumption of patients with mild to moderate AUD was less severely affected by the pandemic^5^ compared to those with severe dependence, who demonstrated a higher resumption of alcohol consumption and greater amounts of consumption^6^. In addition to the severity of AUD, several other factors were found to be associated with increased alcohol consumption, including depressive symptoms, elevated stress levels, job loss, self-reported intensity of stay-at-home orders, and diminished external responsibilities (i.e., working remotely)^4^.

While the global impact of the coronavirus pandemic on addiction does not appear to be significant overall, it shows considerable variation when environmental and contextual factors with respect to individual trajectories are considered^3^. Therefore, this paper focuses on a particularly vulnerable group of patients with AUD, that has not been extensively examined in the current literature. This paper reports on the impact of the pandemic on patients with severe AUD and a complex and challenging clinical profile and a severe drinking history, including multiple inpatient treatments, high levels of daily alcohol consumption and persistent challenges in maintaining abstinence. These patients participated in an outpatient treatment program that is strongly context dependent, involving three weekly supervised administrations of disulfiram (Antabuse). In certain cases, under specific circumstances pertaining to individual patients necessitated a tailored approach. Under such conditions it was sometimes decided to allow patient to self-administer disulfiram (Antabuse) for a designated period. Disulfiram (Antabuse) has been available for over five decades, but its use in the treatment of alcohol addiction has remained limited for various reasons. Disulfiram (Antabuse) is considered an effective treatment option for AUD^7,8^, although the long-term effects on abstinence have not yet been established^9^. The wider clinical use of disulfiram (Antabuse) is significantly impaired by safety concerns due to its unique mechanism of action: disulfiram (Antabuse) blocks the enzyme aldehyde dehydrogenase in the liver. The ingestion of alcohol during treatment results in the accumulation of acetaldehyde, which is likely to result in an aversive reaction, including a fast heart and respiratory rate, facial flushing, nausea, vomiting, and, in the worst case, cardiovascular collapse^9^. However, studies consider disulfiram (Antabuse) to be safe, provided that the intake is supervised and monitored^10^.

The objective of this study is to gain further insight into the impact of restrictions during the Covid-19 pandemic on people with AUD, with a particular focus on patients who are on ongoing long-term therapy with disulfiram (Antabuse). Given the nature of such an intensive therapy, it can be assumed that the retention rate, i.e. participation in therapy is reduced or discontinued during the lockdown period. Retention rate in addiction focused treatment is one of the most established and consistent parameters associated with a favorable treatment outcome^11,12^.

Consequently, our hypothesis is that patients visited the outpatient clinic less frequently during the lockdown period to continue treatment than before and after the lockdown period. In order to investigate the long-term effects of the pandemic, we will analyze longitudinal data on patient participation in therapy in three time periods: baseline, lockdown, and post-lockdown.

## Methods

We examined long-term data from all patients treated with disulfiram (Antabuse) in our outpatient clinic between 2019 and 2022 (n = 45), which we assigned to three time periods. The baseline period (T1) refers to the last 12 months before the first lockdown in Germany (April 2019 to March 2020). The lockdown period (T2) refers to the lockdown months (April to June 2020 and November 2020 to June 2021). The post-lockdown period (T3) refers to the 12 months after the end of the last lockdown (July 2021 to June 2022). The study encompassed three distinct phases, with data collected from the same subjects across these phases, resulting in a dependent sample structure. The statistical parameters reported in the study are a direct consequence of this dependency. To account for this pairwise Wilcoxon tests for dependent samples were conducted to examine differences in the number of patients treated at the outpatient clinic. Given that the time periods varied in length, the frequency of clinic visits was normalized by adjusting for the number of months in each period. The degrees of freedom of the linear trend analysis (df = 33) reflect the total number of months included in the study. The primary focus of the analysis was the comparison of models with consistent versus differing slopes to capture trends over time.

In order to examine group differences with regard to the number of patients treated in the outpatient clinic in the three time periods, pairwise Wilcoxon tests for dependent samples were carried out. With the acknowledgements that the time periods differ in length, the frequency of visits to the outpatient clinic was adjusted for the number of months in the study periods. Additionally, a linear trend analysis was carried out to gain insights into the long-term change in the number of patients treated over the three time periods. This study was approved by the ethics committee (reference number BO-EK-188052024) at the Faculty of Medicine at the Technische Universität Dresden, Germany. All research was performed in accordance with relevant guidelines/regulations and the Declaration of Helsinki. Informed consent was obtained from all participants.

## Results

Table 1 shows sociodemographic and clinical parameters of the participants. The patient cohort comprises 45 participants, with the majority being male, constituting 66.7% (n = 30). The entire patient cohort is of German ethnicity. The average age of the patients is 51.57 years (SD = 11.3). Sixty percent (n = 27) of the patients completed vocational training, while 13.9 percent patients (n = 6) achieved a university degree. For 16.3% (n = 7) of the patients, the highest level of education attained was completed secondary school. Furthermore, the data indicates that 46% (n = 20) of patients were employed. A quarter of the patients were retired or permanently disabled (25.6%, n = 11), while another quarter were unemployed (27.9%, n = 12). Information about the current occupational status was missing for 2 patients.

**Table 1:** Sociodemographic and clinical parameters.

	Mean	Standard deviation	Number	(%)
Age	52	11		
Sex				
Male			30	66.7%
Female			15	33.3%
Marital status				
Single			24	53.3%
Married			8	17.8%
Widowed			2	4.4%
Living separated			11	24.5%
Nationality				
German			45	100.0%
Highest educational qualification achieved				
No school leaving certificate/special school			3	7.0%
Secondary school education			7	16.3%
Vocational training			27	62.8%
University degree or higher			6	13.9%
Occupational status				
Unemployed			12	27.9%
Employed			20	46.6%
Retired or permanently disabled			11	25.6%
Previous medication for reoccurrence prevention				
Naltrexone			27	46.6%
Acamprosate			12	20.7%
Baclofen			3	5.2%
Nalmefene			4	6.9%
None			12	20.7%
Psychatric diagnosis				
Other substance use disorders (excluding nicotine)			45	100%
Psychotic disorders			3	6.7%
Affective disorders			28	62.2%
Neurotic, stress-related, and somatoform disorders			10	22.2%
Personality Disorders			6	13.3%
AD(H)D			1	2.2%
Alcohol parameters				
Age of first consumption in years	14.81	2.54		
Duration of dependency in months	176.98	106.85		
Consumption in g/day before disulfiram (Antabuse)	280.59	157.69		

Regarding marital status, the patients were distributed as follows: 53.3% (n = 24) were single, 17.8% (n = 8) were married, 24.5% (n = 11) were separated, and 4.4% (n = 2) were widowed.

Among the patients diagnosed with psychiatric comorbidities, 62.2% (n = 28) had affective disorders according to ICD-10 (F30-39). Additionally, 22.2% (n = 10) of the patients had neurotic, stress-related, and somatoform disorders (F40-48 ICD-10), while 13.3% (n = 6) were diagnosed with personality and behavioral disorders (F60-69 ICD-10). A small percentage of 6.7% (n = 3) presented schizophrenia, schizotypal, and delusional disorders (F20-29 ICD-10), and 2.2% (n = 1) were diagnosed with behavioral and emotional disorders with onset in childhood and adolescence (F90-98 ICD-10).

Before disulfiram (Antabuse) initiation, naltrexone was administered to 46.6% (n = 27), acamprosate to 20.7% (n = 12), nalmefene to 6.9% (n = 4), and baclofen to 5.2% (n = 3) of the patients. Approximately one-fifth of the patients, 20.7% (n = 12), reported not receiving any prior medication for return to alcohol use prevention.

The average age of first alcohol consumption for the patients was 14.81 years (SD = 2.54). The duration of dependency averaged176.98 months (SD = 106.85) resp. 4.75 years (SD = 8.90). The mean daily alcohol consumption before disulfiram (Antabuse) initiation was 280.59 g per day (SD = 157.69).

**Fig. 1:**
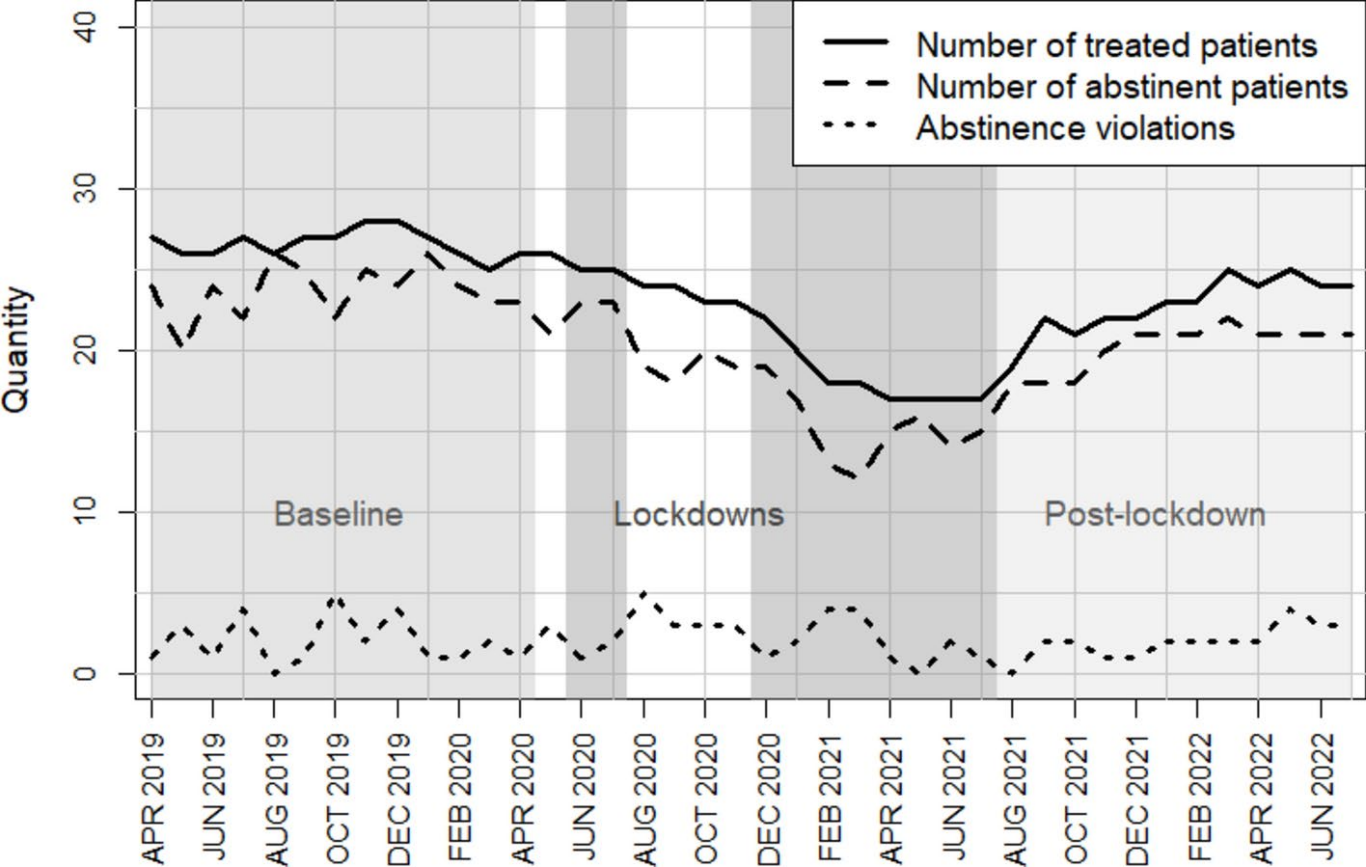
Number of treated patients, abstinent patients and abstinence violations during the Covid pandemic.

On average, 26.67 patients were treated in the outpatient clinic in period T1, 20.82 in T2 and 22.25 in T3, which already suggests differences in the retention rate between the periods considered (Fig. 1). To test the hypothesis that the frequency of visits to the outpatient clinic to continue therapy (relative to the length of the time periods) differs in the three time periods, three pairwise comparisons were carried out using Wilcoxon tests. These showed a significant difference between T1 and T2 (*V* = 200, *p* = 0.031, *r* = 0.32), but no significant differences between T2 and T3 (*V* = 259.5, *p* = 0.357) or between T1 and T3 (*V* = 462.5, *p* = 0.314). It can therefore be shown that the mean number of ambulance visits differs between the period before and during the lockdowns.

**Fig. 2:**
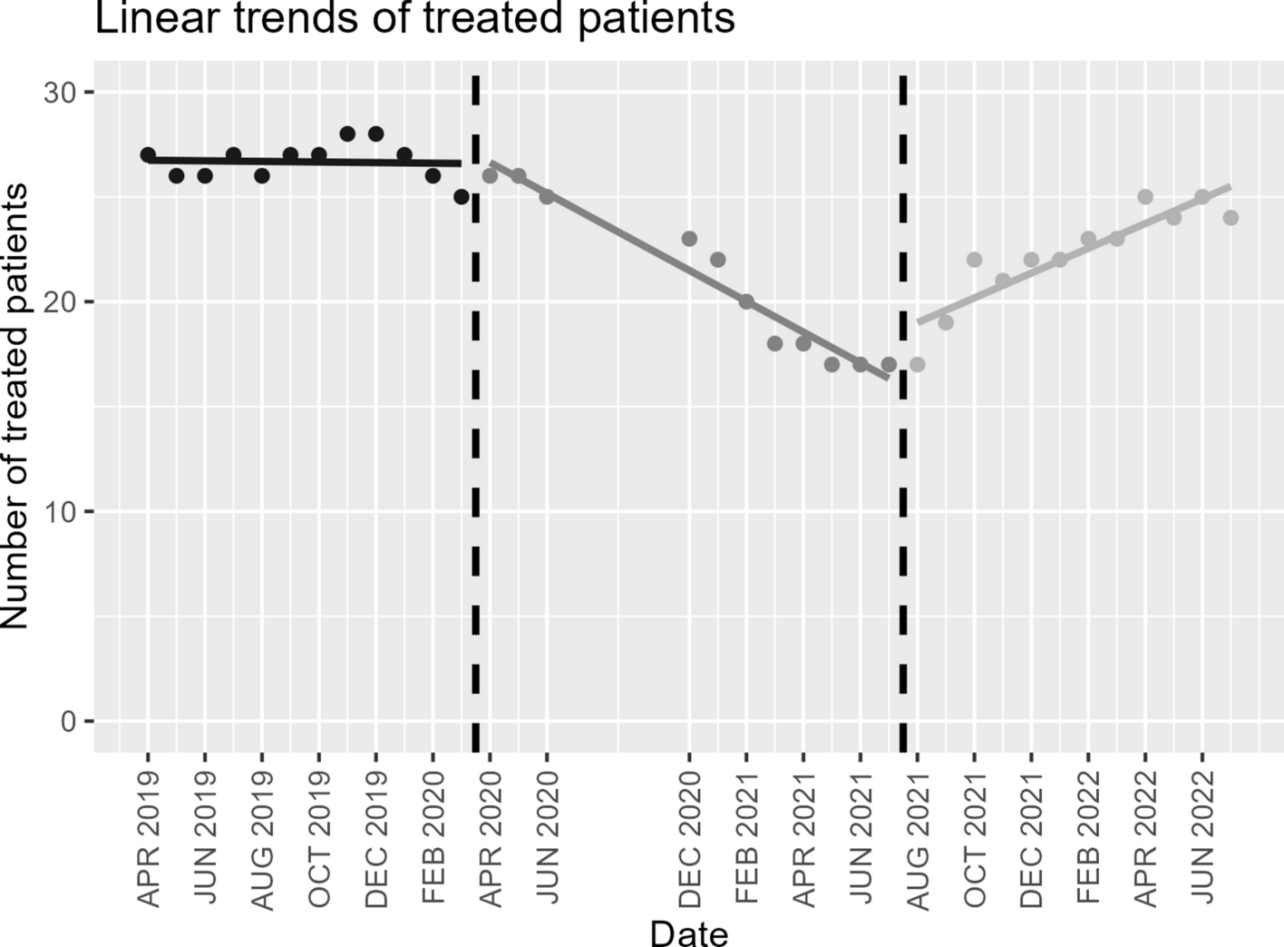
Linear trend analysis for the number of treated patients during the Covid pandemic.

To investigate the differences between the periods in more detail and take the long-term data into account, a linear trend analysis was carried out (Fig. 2). For comparison purposes, a reference model was first calculated in which a common linear trend is assumed over all time periods. The reference model examines the effect of the predictor date on the dependent variable number of patients. The fitted regression model of the reference model was: (Number of treated patients) = 29.32–0.17 * (date). It was found that date predicted the number of patients (*t* = − 4.07, *p* < 0.001). The overall regression was statistically significant (*R*^2^ = 0.334, *F* (1, 33) = 16.55, *p* < 0.001). However, the model only accounts for 33.4% of the variability of the dependent variable in the data set,

Assuming that there were different trends in each of the periods under consideration, a second linear regression model was carried out assuming different increases. Accordingly, the predictors were the date and threshold values, and the dependent variable was still the number of patients treated. The fitted regression model was: (Number of treated patients) = 26.97 + 20.26 * (threshold 1) – 33.38 * (threshold 2) – 0.01 * (date) – 0.72 * (threshold 1 * date) – 0.60 * (threshold 2 * date). The overall regression was statistically significant (*R*^2^ = 0.934, *F* (5, 29) = 81.47, *p* < 0.001) and accounted with 93.4% for considerably more of the variability of the dependent variable in the data set. All predictors, except date, and all interaction effects showed a significant influence on the dependent variable (all *p* < 0.001).

The distinctly better fit of the second model shows that a model assuming different trends between the time periods can explain the data considerably better, which supports the claim that during the lockdowns there was a change in the trend with regard to the retention rate of the disulfiram (Antabuse) therapy. These different trends are also visible graphically (Fig. 2).

## Discussion

The present study suggests that the coronavirus pandemic had a negative impact on the treatment trajectories of patients with severe AUD who were in an intensive outpatient treatment program with disulfiram (Antabuse) prescription. Prospective long-term monitoring (2019–2022) of clinical routine parameters revealed several meaningful changes especially in the patients’ retention rate. During April 2020 and July 2021, there were increased numbers of treatment discontinuations. After August 2021, the number of treated patients slowly recovered. Our results indicated that patients visited the outpatient clinic less frequently during the lockdown period to continue treatment than before the lockdown period. However, Figs. 1 and 2 indicate a gradual return in the number of treated patients to pre-lockdown levels, following the end of the lockdown showing that the number of treated patients appears to be slowly returning to pre-lockdown levels once the lockdown ended.

Concurrently, linear trend analysis revealed that the lockdown period in T2 was accompanied by a pronounced shift in the trend, which then reversed again over T3. The retention rate decreased over T2, while the number of patients in the program increased again in T3. These changes occurred in close temporal relation with the pandemic restrictions and could therefore suggest that the Covid pandemic and its related restriction might have had an impact on patients with severe AUD.

We aim to contextualize this potential interpretation within the broader scope of existing research on the relationship between mental health and the consequences of the pandemic.

In the context of the restrictions imposed in Germany, it is noteworthy that there was no explicit prohibition on visiting health-related services. Numerous additional requirements were implemented, including hygiene protocols, restrictions on patient capacity, and social distancing measures. Our findings are in line with other developments that have been observed in the context of the coronavirus pandemic in almost all medical areas, including psychiatric care. For example, the utilization of individual and group psychotherapy and opioid substitution treatment declined significantly during the first half of 2020 in Germany^13^. Furthermore, the number of alcohol-related inpatient hospital treatments in Germany was 17% lower in 2021 than in 2019^14^.

With regard to patients who received disulfiram (Antabuse) treatment for AUD, Fortingiuerra et al. and Balhara et al. both reported a decrease in disulfiram (Antabuse) prescriptions among patients with severe dependence during the pandemic^6,15^. The results from Balhara et al. demonstrate a significant decline of 60%, suggesting that the strict lockdown measures implemented in India from March 2020, where alcohol was considered a non-essential item during the pandemic, may have had a profound impact^15^. These findings are supported by a reduction in the number of outpatients treated in our program, which decreased by approximately 35% from an average of 26.67 to 20.82 patients. This also resulted in a markedly decreased prescription of disulfiram (Antabuse). Our study thus joins the ranks of studies that find evidence in favor of the hypothesis that patients experience a deterioration in alcohol consumption during the period of lockdown^16^. We believe that our very close-knit treatment concept with up to three weekly consultations was particularly susceptible to pandemic-related barriers.

Our sample was characterized by high rates of comorbidities the majority of which were affective disorders. These comorbidities might have influenced patients’ capacity to attend healthcare appointments, particularly during the stress and uncertainty of the pandemic. For instance, individuals suffering from depression or anxiety may have experienced heightened trepidation regarding attending medical visits, even when deemed essential, or may have been more adversely affected by the disruptions caused by the pandemic. Future studies to examine the interplay between alcohol use disorder and comorbidities in influencing healthcare-seeking behavior, particularly during crisis situations such as the Corona Pandemic are necessary.

Potential barriers to treatment include fear of contracting COVID-19 during travel, lack of transportation, reduced services provided by healthcare facilities, and restrictions imposed by governmental agencies. These findings are consistent with those of Fortingiuerra et al. and Balhara et al., which indicate that patients with severe AUD were particularly impacted by lockdown restrictions^6,15^. A particularly concerning observation is that the number of treatment cases did not normalize to pre-pandemic levels even by 2023. This is emphasized by data from healthcare providers, which indicates that there was an increasing number of AUD cases, but at the same time a decreasing number of addiction-specific inpatient treatments during the pandemic^17^.

The pandemic may have resulted in individuals becoming less willing to seek professional help for health issues. This is an unfortunate development in light of the findings of our secondary study, which indicated that among patients who continued despite the barriers in the disulfiram-assisted program, there was no increase in the number of abstinence violations (Fig. 1). Therefore, even under pandemic conditions, disulfiram-assisted treatment appears to be effective for patients with severe AUD.

The findings presented herein contribute to a deeper understanding of the impact of environmental effects on disulfiram (Antabuse) therapy, partially confirming established knowledge in the scientific literature. This understanding is crucial for adapting clinical practices during crises and improving the care of individuals with alcohol dependence. In some promising examples, the restrictions have already been successfully addressed by offering telemedical consultations^18^.

Future studies should conduct detailed investigations of the long-term effects of pandemics on disulfiram (Antabuse) therapy, as well as the long-term efficacy of this therapy on preventing reoccurrence. This expanded knowledge base would be beneficial for the continuous and consistent treatment of AUD during unforeseen environmental circumstances like pandemics.

## Limitations

While our results might provide valuable insights, several limitations must be acknowledged. Firstly, the relatively small sample size (n = 45) limits the statistical power and generalizability of the findings. Consequently, the results of the trend analysis should be interpreted with caution until they are validated by larger samples. Secondly, as a retrospective study, it is not possible to establish causality between the pandemic and reduced retention rate. The observed changes may have been also influenced by other factors, such as psychiatric comorbidities, personal health concerns or societal disruptions.

Thirdly, the retention rate is an outcome parameter established in the field of addiction^11,12^, but it does not allow for differentiated outcome analyses. In this respect, it should be noted that no standardized assessment of additional outcome criteria, such as 'AUD status (i.e., in remission or not), self-rated health, or quality of life, was conducted.

While this study provides important insights into the impact of the pandemic on healthcare utilization among patients with severe AUD, these limitations suggest the need for further research. Further studies should incorporate larger, more diverse samples and include different patient outcome measures, to better understand how global health crises affect healthcare access and patient well-being.

## Data Availability

All data generated or analysed during this study are included in this published article (and its Supplementary Information files).

## References

[1] Pavarin, R. M. et al. COVID-19-related death in patients with alcohol or substance use disorders. *Eur. Addict. Res.* **29**, 67–70 (2023). 36450270 10.1159/000527542 PMC9892992

[2] Friske, M. M. et al. Chronic alcohol intake regulates expression of SARS-CoV2 infection-relevant genes in an organ-specific manner. *Alcohol Clin. Exp. Res.* **47**, 76–86 (2023). 10.1111/acer.14981 36774629

[3] Friske, M. M. & Spanagel, R. Chronic alcohol consumption and COVID-19 infection risk: A narrative review. *Alcohol Clin. Exp. Res.* **47**, 629–639 (2023). 10.1111/acer.15041 36851826

[4] Acuff, S. F., Strickland, J. C., Tucker, J. A. & Murphy, J. G. Changes in alcohol use during COVID-19 and associations with contextual and individual difference variables: A systematic review and meta-analysis. *Psychol. Addict. Behav.* **36**, 1–19 (2022). 34807630 10.1037/adb0000796 PMC8831454

[5] Deeken, F. et al. Patterns of alcohol consumption among individuals with alcohol use disorder during the COVID-19 pandemic and lockdowns in Germany. *JAMA Netw. Open* **5**, e2224641 (2022). 35913741 10.1001/jamanetworkopen.2022.24641 PMC9344361

[6] Fortinguerra, F., Pierantozzi, A. & Trotta, F. The use of medications approved for alcohol use disorders in Italy. *Front. Public Health* **11**, 1110435 (2023). 36875354 10.3389/fpubh.2023.1110435 PMC9975714

[7] Suh, J. J., Pettinati, H. M., Kampman, K. M. & O’Brien, C. P. The status of disulfiram: A half of a century later. *J. Clin. Psychopharmacol.* **26**, 290–302 (2006). 16702894 10.1097/01.jcp.0000222512.25649.08

[8] Skinner, M. D., Lahmek, P., Pham, H. & Aubin, H.-J. Disulfiram efficacy in the treatment of alcohol dependence: A meta-analysis. *PLoS ONE* **9**, e87366 (2014). 24520330 10.1371/journal.pone.0087366 PMC3919718

[9] Jørgensen, C. H., Pedersen, B. & Tønnesen, H. The efficacy of disulfiram for the treatment of alcohol use disorder. *Alcohol Clin. Exp. Res.* **35**, 1749–1758 (2011). 21615426 10.1111/j.1530-0277.2011.01523.x

[10] Jonas, D. E. et al. Pharmacotherapy for adults with alcohol use disorders in outpatient settings: A systematic review and meta-analysis. *JAMA* **311**, 1889–1900 (2014). 24825644 10.1001/jama.2014.3628

[11] Dacosta-Sánchez, D., González-Ponce, B. M., Fernández-Calderón, F., Sánchez-García, M. & Lozano, O. M. Retention in treatment and therapeutic adherence: How are these associated with therapeutic success? An analysis using real-world data. *Int. J. Methods Psychiatr. Res.* **31**, e1929 (2022). 35765238 10.1002/mpr.1929 PMC9720222

[12] Ball, S. A., Carroll, K. M., Canning-Ball, M. & Rounsaville, B. J. Reasons for dropout from drug abuse treatment: Symptoms, personality, and motivation. *Addict. Behav.* **31**, 320–330 (2006). 15964152 10.1016/j.addbeh.2005.05.013

[13] Liu, S., Heinz, A., Haucke, M. N. & Heinzel, S. Globale auswirkungen der COVID-19-pandemie auf die versorgung von Menschen mit psychischen Erkrankungen. *Nervenarzt* **92**, 556–561 (2021). 33575836 10.1007/s00115-021-01068-2 PMC7877534

[14] Landesbetrieb IT.NRW Statistik und IT-Dienstleistungen. NRW: Über 70 Prozent der alkoholbedingten Krankenhausbehandlungen und Sterbefälle betrafen 2021 Männer. https://www.it.nrw/nrw-ueber-70-prozent-der-alkoholbedingten-krankenhausbehandlungen-und-sterbefaelle-betrafen-2021 (2023).

[15] Balhara, Y. P. S., Kattula, D., Singh, S., Chukkali, S. & Bhargava, R. Impact of lockdown following COVID-19 on the gaming behavior of college students. *Indian J. Public Health* **64**, S172–S176 (2020). 32496250 10.4103/ijph.IJPH_465_20

[16] Schecke, H., Bohn, A., Scherbaum, N. & Mette, C. Alcohol use during COVID-19 pandemic on the long run: Findings from a longitudinal study in Germany. *BMC Psychol.* **10**, 266 (2022). 36376933 10.1186/s40359-022-00965-8 PMC9661459

[17] BARMER. BARMER-Analyse: Rund 1,5 Millionen Menschen alkoholabhängig|BARMER. https://www.barmer.de/presse/presseinformationen/pressearchiv/barmer-analyse-rund-1-5-millionen-menschen-alkoholabhaengig-1251732 (2024).

[18] Leibowitz, A. et al. A telemedicine approach to increase treatment of alcohol use disorder in primary care: A pilot feasibility study. *J. Addict. Med.* **15**, 27–33 (2021). 32467415 10.1097/ADM.0000000000000666 PMC7704783

[19] Spanagel, R. et al. The ReCoDe addiction research consortium: Losing and regaining control over drug intake-Findings and future perspectives. *Addict. Biol.* **29**, e13419 (2024). 38949209 10.1111/adb.13419 PMC11215792

